# Multidimensional evaluation of 3.0T HR-MRI, ultrasound imaging, and GATA3 protein expression in breast cancer, and their prognostic analysis

**DOI:** 10.3389/fonc.2025.1587243

**Published:** 2025-08-13

**Authors:** Yang Bai, Ting Xie, Xiangyang Mo, Yong Luo, Man Chen

**Affiliations:** ^1^ The Second Affiliated Hospital, Department of Ultrasound Medicine, Hengyang Medical School, University of South China, Hengyang, Hunan, China; ^2^ The Second Affiliated Hospital, Department of Emergency, Hengyang Medical School, University of South China, Hengyang, Hunan, China; ^3^ The Affiliated Nanhua Hospital, Department of Emergency, Hengyang Medical School, University of South China, Hengyang, Hunan, China

**Keywords:** high-resolution magnetic resonance imaging, ultrasound, GATA3, breast cancer, diffusion tensor imaging

## Abstract

**Objective:**

To evaluate the diagnostic value of 3.0T high-resolution magnetic resonance imaging (3.0T HR-MRI), ultrasound imaging, and GATA3 protein expression in breast cancer (BC) and their prognostic implications.

**Methods:**

A retrospective analysis of 143 BC patients was conducted. All patients underwent preoperative 3.0T HR-MRI and ultrasound examinations. Diffusion tensor imaging (DTI) parameters, including mean diffusivity (MD), fractional anisotropy (FA), radial diffusivity (Dr), and axial diffusivity (Da), were assessed. Ultrasound features such as tumor morphology, vascularity, and posterior acoustic characteristics were analyzed. Immunohistochemistry was performed to detect GATA3 expression, and correlations with imaging parameters and prognosis were evaluated.

**Results:**

GATA3 expression was significantly associated with BC pathological subtypes ( *x* ^2^= 26.59, P < 0.0001). Compared with the GATA3-negative group, GATA3-positive tumors exhibited lower FA but higher MD, Dr, and Da values (P < 0.05), suggesting more preserved tissue structure. Ultrasound analysis showed that GATA3-positive tumors had less vascularity, posterior attenuation, and irregular margins (P < 0.05). Poor prognosis was associated with higher FA and lower MD, Dr, and Da values (P < 0.0001), as well as more aggressive ultrasound features. ROC analysis demonstrated superior prognostic performance when combining GATA3 expression, MRI, and ultrasound parameters (AUC = 0.9695, sensitivity = 83.54%, specificity = 96.88%).

**Conclusion:**

3.0T HR-MRI and ultrasound provide complementary insights into BC characteristics. GATA3 expression is associated with better prognosis, and their combined analysis enhances diagnostic accuracy and prognostic evaluation.

## 1 Introduction

Breast cancer (BC) is one of the most common malignancies among women worldwide, with its incidence steadily rising in recent years (1). Statistics show that BC has become a leading cause of cancer-related mortality among women globally, particularly in developed countries and regions, where the incidence remains high (2). The onset and progression of BC are influenced by various factors, including genetics, environment, hormone levels, and lifestyle (3). Despite significant advances in early diagnosis and treatment, the heterogeneity and complexity of its different molecular subtypes present ongoing challenges in accurately assessing and predicting tumor progression (4).

Currently, the diagnosis of BC in clinical practice mainly relies on imaging examinations, histopathological analysis, and molecular marker detection (5). Imaging techniques include mammography, ultrasound (US), and magnetic resonance imaging (MRI), all of which play a critical role in detecting tumor size, shape, boundaries, and internal structure (6). However, the accuracy and sensitivity of a single imaging modality can be limited in certain cases. For example, US may miss small lesions or fail to detect abnormalities in patients with complex glandular structures, while mammography tends to have a lower detection rate in dense breast tissue (7). In comparison, 3.0T HR-MRI offers higher soft tissue resolution, enabling more precise visualization of the subtle structure and diffusion characteristics of breast tumors. Emerging techniques such as Diffusion Tensor Imaging (DTI) are particularly valuable for assessing the microstructure and biological behavior of tumor tissue (8).

Among molecular markers in BC, GATA3 expression has garnered significant attention in recent years. GATA3, a member of the GATA family of transcription factors, plays a crucial regulatory role in the differentiation of breast epithelial cells (9). Studies have shown that GATA3 expression is closely associated with molecular subtypes, clinicopathological features, and prognosis of BC. Specifically, high GATA3 expression in Luminal A and Luminal B subtypes is often correlated with a favorable prognosis, whereas low or absent expression may indicate higher tumor aggressiveness (10). Consequently, GATA3 expression serves as an important marker for molecular subtyping and prognosis in BC.

Although imaging examinations and molecular marker detection both have their advantages in diagnosing and prognosticating BC, the diagnostic efficacy of a single approach remains limited. 3.0T HR-MRI can accurately evaluate tumor size, shape, and boundaries, but it lacks the ability to directly reflect the molecular characteristics and biological behavior of the tumor (11). On the other hand, molecular markers such as GATA3 provide insights at the molecular level, but these tests typically require invasive tissue sampling and may not adequately capture the tumor’s spatial heterogeneity in real-time (12). Therefore, combining high-resolution imaging with molecular marker detection could offer a more comprehensive diagnostic approach, enhancing diagnostic accuracy and improving the precision of prognosis predictions.

Based on this, the present study conducted a multi-dimensional diagnostic and prognostic evaluation of BC patients by analyzing the associations among 3.0T HR-MRI, ultrasound imaging features, and GATA3 protein expression levels. The study aims to provide a more comprehensive and precise diagnostic foundation for clinical practice, optimize individualized treatment strategies for BC, and improve the accuracy of diagnosis and the scientific rigor of prognosis management, thereby offering solid theoretical support for clinical application.

## 2 Materials and methods

### 2.1 General data

A retrospective analysis was conducted on the clinical data of 143 female BC patients treated at our hospital between June 2020 and April 2023. Pathological tissue specimens were obtained either through surgical resection or ultrasound-guided biopsy, followed by immunohistochemistry to determine GATA3 protein expression.

Inclusion criteria: Patients who were confirmed as BC by surgery and had complete immunohistochemical data. All patients underwent 3.0T HR-MRI and ultrasound examinations within two weeks before surgery.

Exclusion criteria: Patients with incomplete clinical or pathological data, unclear imaging, or undefined parameters on 3.0T HR-MRI and ultrasound. Additionally, patients with multiple cancer foci, those not newly diagnosed, or those who had undergone adjuvant therapy, as well as patients with a history of other malignant tumors, and patients who did not consent to participate in the study, were excluded. A total of 143 patients were finally included in the study, with an average age of (55.30 ± 9.86) years.

### 2.2 Methods

#### 2.2.1 Bioinformatics analysis

Relevant datasets, including GSE15852, GSE45827, and GSE0014, were obtained from the Gene Expression Omnibus (GEO) database to identify differentially expressed genes (DEGs) in BC by comparing BC samples with normal controls. KM-plotter was used to analyze survival data of differential genes. The Cancer Genome Atlas (TCGA) database was used to analyze the expression of GATA3 across various cancer types.

#### 2.2.2 Clinical data

**3.0T High-Resolution Magnetic Resonance Imaging:** The imaging was performed using a GE 3.0T HMR scanner (GE Healthcare, USA) equipped with a 16-channel bilateral phased-array breast coil. The T1-weighted imaging (T1WI) sequence parameters were as follows: TR = 750.00 ms, TE = 9.00 ms, FOV = 14 mm, matrix = 512 × 512, slice thickness/spacing = 1 mm/0 mm, and imaging time = 110 s. The T2-weighted imaging (T2WI) sequence parameters were: TR = 2250.00 ms, TE = 80.00 ms, FOV = 14 mm, matrix = 512 × 512, slice thickness/spacing = 1 mm/0 mm, and imaging time = 138 s. For diffusion tensor imaging (DTI), the scan parameters were: 5 b-values (0, 500, 1000, 1500, and 2000 s/mm²), TR = 3900 ms, TE = 94 ms, FOV = 14 mm, matrix = 98 × 160, slice thickness/spacing = 1 mm/0 mm, and imaging time = 540 s. Upon completion of the scanning, the images were uploaded to a post-processing workstation for analysis by two radiologists. Key diffusion metrics, including mean diffusivity (MD), fractional anisotropy (FA), radial diffusivity (Dr), and axial diffusivity (Da), were measured and recorded. Each measurement was repeated three times, and the average value was taken for further analysis.

**Ultrasound Examination:** Ultrasound was performed using the CE LOGIQ E9 color Doppler ultrasound diagnostic instrument with a high-frequency linear array probe (frequency range: 814 MHz). The patient was positioned supine to expose both breasts and axillae, scanning included the breasts and draining lymph nodes. Static images of the longest axial and transverse sections of the lesion and dynamic videos of color Doppler flow signals were saved. Preoperative ultrasound was conducted by a physician with over seven years of experience to ensure image consistency. The selected two-dimensional ultrasound features included the longest lesion diameter, lesion boundary (clear/unclear), aspect ratio (<1/≥1), shape (regular/irregular), and posterior acoustic features (normal/attenuated/enhanced). Blood flow intensity was semi-quantitatively categorized into four levels: grade 0 (no blood flow), grade I (1–2 dot or short rod-shaped blood flow signals in the lesion), grade II (3–4 dot or rod-shaped blood flow signals or blood flow signals exceeding the lesion radius), and grade III (more than 4 large blood flow signals or the presence of network-forming blood flow). Grades 0-I were defined as low blood supply lesions, while grades II-III were defined as high blood supply lesions. Data were double-blind reviewed by two deputy chief sonographers, and disagreements were resolved through consultation.

**Immunohistochemistry (IHC):** GATA-3-positive cells exhibited staining localized to the nucleus, appearing as yellow-brown or dark-brown coloration. Any amount of nuclear staining in tumor cells was considered positive.

**Definition of Recurrence**: All patients were followed up after surgery via telephone, WeChat, or outpatient visits until December 2024. The definition of recurrence was based on the European Society for Medical Oncology (ESMO) Clinical Practice Guidelines (2019 edition) (13), including local recurrence, regional lymph node recurrence, or distant metastasis. All recurrence events were confirmed by clinical, radiological, and/or pathological evidence. Based on recurrence status during the follow-up period, patients were classified into a good prognosis group (no recurrence) and a poor prognosis group (with recurrence) for subsequent prognostic analysis.

### 2.3 Statistical methods

Statistical analysis was performed using SPSS 22.0. Measurement data following a normal distribution were expressed as mean ± standard deviation ( *x̄* ± s) and analyzed using the t-test. Non-normally distributed data were expressed as median (Q_min_, Q_max_) and analyzed using the rank-sum test. Categorical data were expressed as n (%) and analyzed using the chi-square test. The diagnostic value of combined 3.0T HR-MRI, ultrasound, and GATA3 was evaluated using ROC curves. P < 0.05 was considered statistically significant.

## 3 Results

### 3.1 Screening of DEGs in breast cancer-related GEO datasets

**Figure 1:**
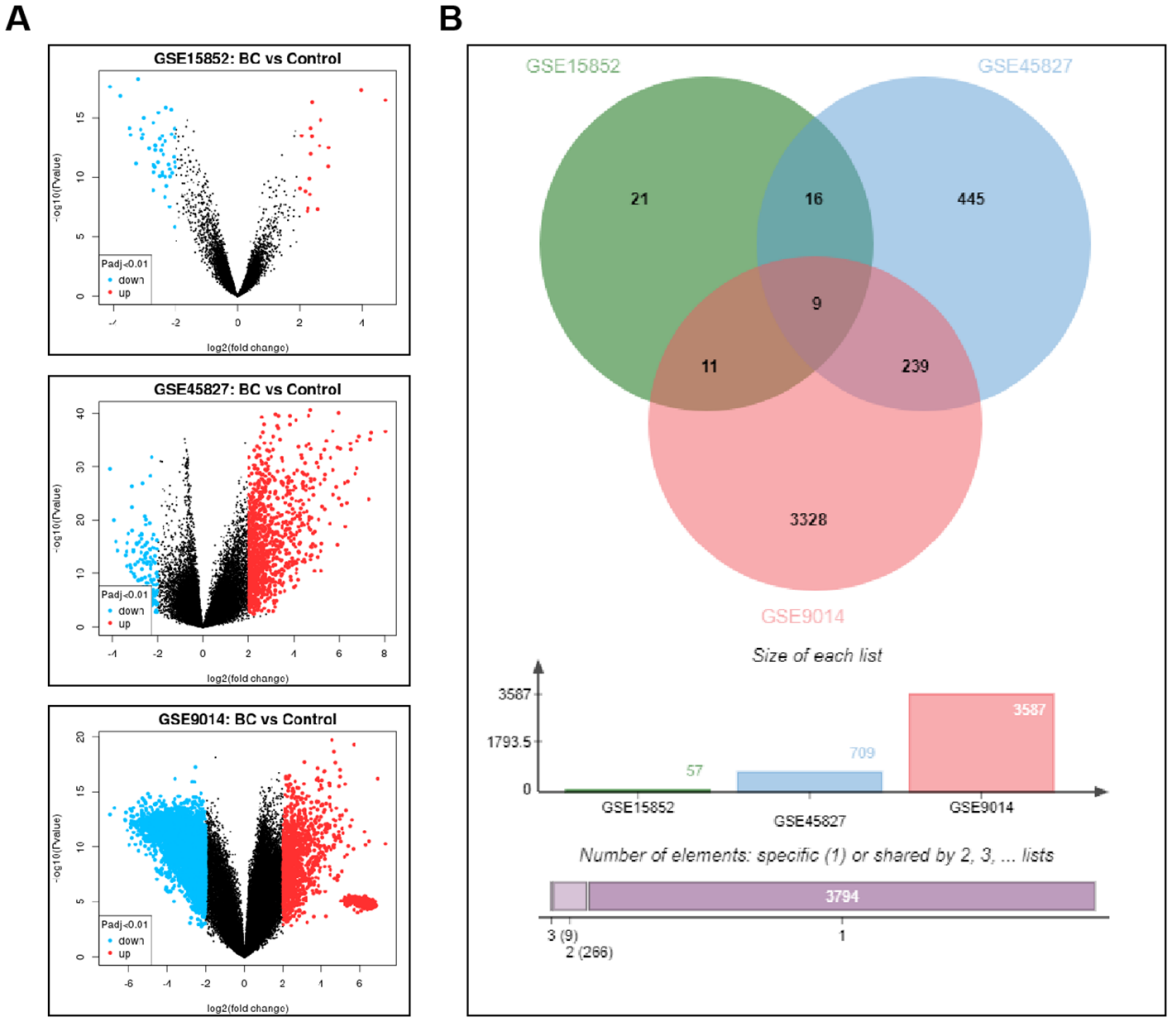
Screening of Differentially Expressed Genes in BC-Related GEO Datasets. **(A)** The volcano plot illustrates the gene expression differences between BC samples and control groups in the GSE15852, GSE45827, and GSE9014 datasets. **(B)** The Venn diagram displays the common differentially expressed genes identified across all three datasets.

This study conducted an analysis of DEGs in BC-related GEO datasets, as shown in **Figure 1A**. The datasets selected for the analysis included GSE15852, GSE45827, and GSE9014, where gene expression differences between BC samples and normal control groups were compared to identify significantly differentially expressed genes. The Venn diagram (**Figure 1B**) displays the DEGs that were consistently found across all three datasets, with a total of nine genes commonly identified, including RBP4, PCOLCE2, RGS1, GATA3, TF, CAV1, AKR1C3, MFAP5, and IGLV1-44. These genes exhibited significant differential expression in BC samples, suggesting their potential roles in the occurrence and progression of BC.

### 3.2 GATA3 expression analysis in BC prognosis and various cancer types

**Figure 2:**
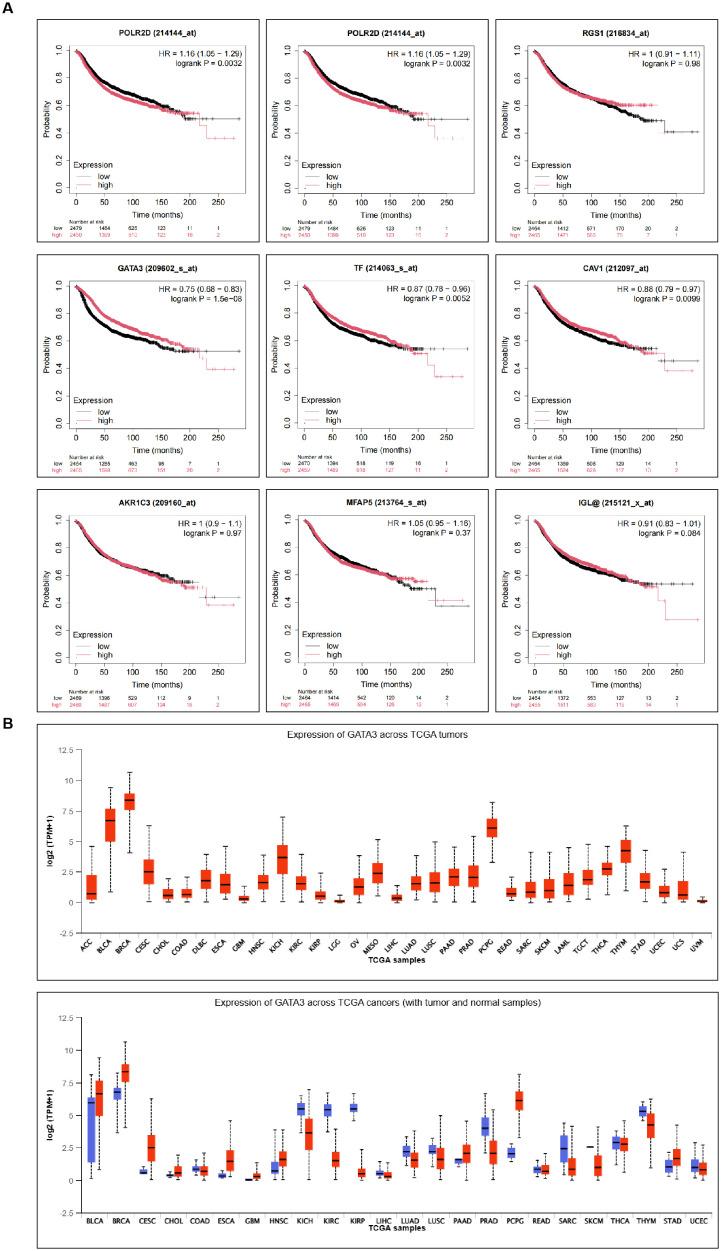
Prognostic Relevance of Differential Genes in BC and Expression Analysis of GATA3 Across Various Cancer Types. **(A)** Kaplan-Meier plotter analysis shows the survival curves of the nine differentially expressed genes in BC patients; **(B)** TCGA database analysis illustrates the expression levels of GATA3 across multiple cancer types.

KM-plotter analysis of survival data for the 9 differentially expressed genes revealed a significant correlation between GATA3 and the prognosis of BC patients (**Figure 2A**). Using BRCA data from the TCGA database, further analysis of GATA3 expression across multiple cancer types showed significant differences, with a notable upregulation in BC patients (**Figure 2B**). Therefore, this study focuses on GATA3 to explore its potential role in BC.

### 3.3 GATA3 expression in BC patients with different clinicopathological characteristics

Among the 143 BC patients, 68 cases showed positive GATA3 expression, while 75 cases were negative. The general data indicated that GATA3 expression was significantly associated with the pathological subtype, TNM stage, lymph node metastasis, recurrence, and survival status of BC (P<0.05), but it showed no significant correlation with age, menopausal status, or pathological type (P>0.05), as shown in **Table 1**.

**Table 1:** GATA3 expression in different clinicopathological characteristics ( *x̄* ± s, n%).

Characteristic			GATA3			
			Negative (n=75)	Positive (n=68)	t/*x* ^2^ Value	P Value
Age (year)			56.51 ± 8.88	56.16 ± 9.30	0.23	0.82
Menopausal Status	Premenopausal	62	31 (41.33%)	31 (45.59%)	0.26	0.61
	Postmenopausal	81	44 (58.67%)	37 (54.41%)		
Pathological Subtype	Luminal A	53	18 (24.0%)	35 (51.47%)	26.59	<0.0001
	Luminal B	38	15 (20.0%)	23 (33.82%)		
	HER-2 Positive	33	27 (36.0%)	6 (8.82%)		
	TNBC	19	15 (20.0%)	4 (5.88%)		
Pathological Type	Invasive	63	44 (58.67%)	19 (27.94%)	2.81	0.09
	Non-invasive	80	31 (41.33%)	49 (72.06%)		
TNM Stage	I-II Stage	78	32 (42.67%)	46 (67.65%)	8.98	0.003
	III-IV Stage	65	43 (57.33%)	22 (32.35%)		
Lymph Node Metastasis	Yes	78	48 (64.0%)	30 (44.12%)	5.69	0.02
	No	65	27 (36.0%)	38 (55.88%)		
Recurrence	Yes	44	29 (38.67%)	15 (22.06%)	4.62	0.03
	No	99	46 (61.33%)	53 (77.94%)		
Survival status	Yes	128	63 (84.0%)	65 (95.59%)	5.10	0.02
	No	15	12 (16.0%)	3 (4.41%)		

### 3.4 Correlation between 3.0T HR-MRI parameters and GATA3 protein expression

The results showed that in BC patients, 3.0T HR-MRI parameters revealed significant differences between the GATA3-positive and GATA3-negative groups. The FA parameter decreased in the GATA3-positive group, while the Da, MD, and Dr parameters increased, with statistically significant differences (P < 0.05). These findings suggest that tumors in GATA3-positive patients exhibit relatively intact tissue structure and reduced anisotropy of water molecule diffusion, indicating a potentially better prognosis. See **Table 2**.

**Table 2:** Correlation between 3.0T HR-MRI parameters and GATA3 expression ( *x̄* ± s).

Parameter	GATA3			
	Negative	Positive	T Value	P Value
FA	0.51 ± 0.08	0.38 ± 0.07	10.00	<0.0001
DA	2.01 ± 0.04	2.10 ± 0.08	9.26	<0.0001
MD	1.20 ± 0.05	1.34 ± 0.12	9.46	<0.0001
Dr	1.08 ± 0.09	1.20 ± 0.11	11.04	<0.0001

Imaging Features of a Typical Case of Breast Cancer Using 3.0T HR-MRI

**Figure 3:**
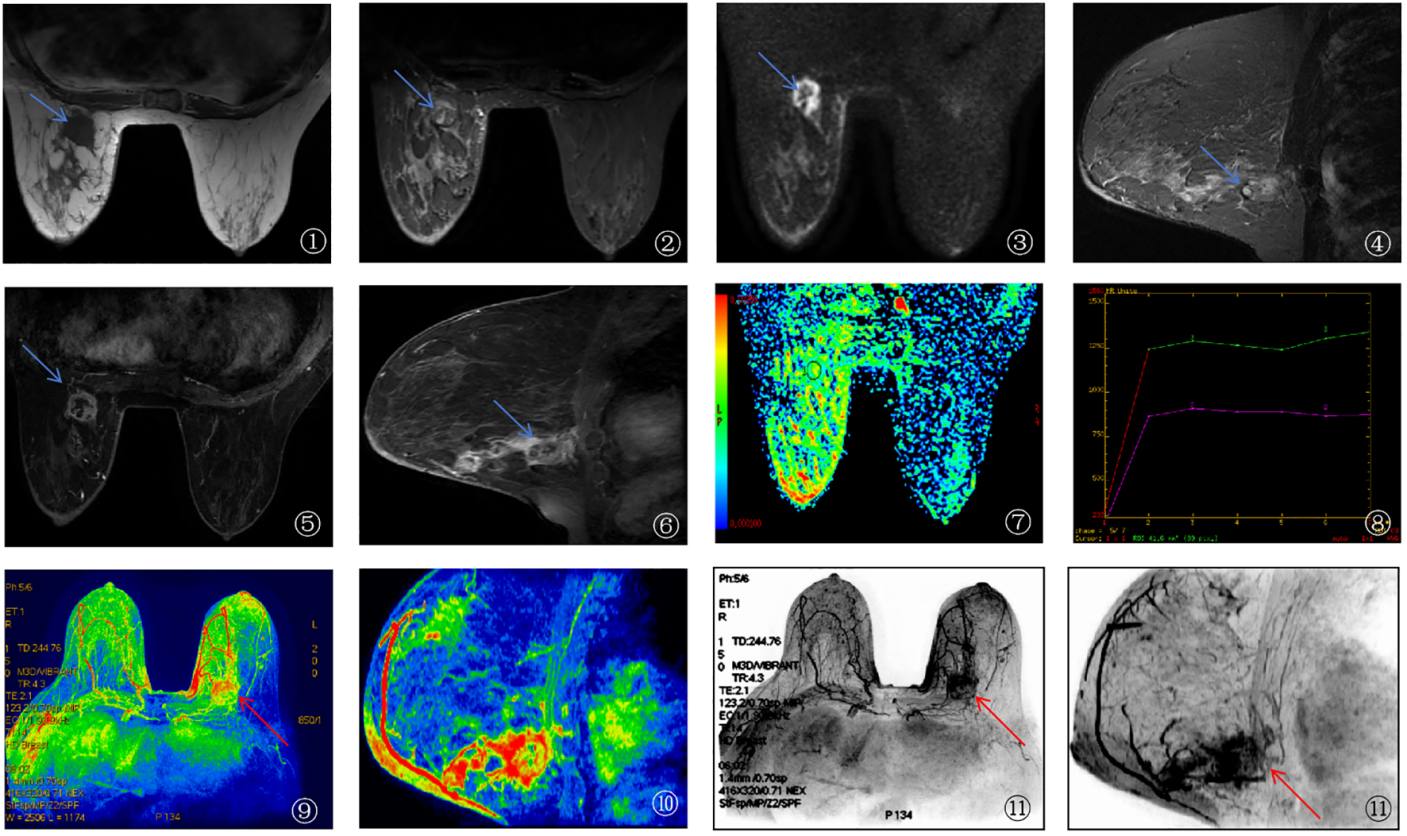
3.0T HR-MRI imaging findings of breast cancer patients.

A 62-year-old female patient diagnosed with breast cancer underwent 3.0T HR-MRI. The imaging findings are detailed as follows: ① The T1WI image showed the tumor (blue arrow) presenting a slightly hypointense signal compared to the surrounding normal glandular tissue, indicating lower density of the tumor; ② The fat-suppressed T2-weighted image (T2-FS) showed the tumor (blue arrow) presenting a mixed hyper- and hypointense signal, suggesting heterogeneity within the tumor; ③, ④ DWI (b=1000 s/mm²) demonstrated that the tumor (blue arrow) exhibited a marked hyperintense signal, indicating high cellular density and restricted water molecular diffusion; ⑤, ⑥ Dynamic contrast-enhanced imaging (DCE-MRI) showed irregular margins and significant enhancement of the tumor, indicating rich vascularization; ⑦ The pseudo-colored ADC map (apparent diffusion coefficient) showed the tumor region of interest (ROI) as relatively hypointense, further indicating high cellular density; ⑧ The time-signal intensity curve (TIC) demonstrated that the tumor ROI, outlined by the green circle, predominantly followed a plateau-outflow enhancement pattern, suggesting rapid washout after enhancement; ⑨, ⑩ The pseudo-colored MIP image and ⑪ the conventional MIP image showed the lesion with high perfusion characteristics, and the surrounding area exhibited increased and aggregated vascular structures (red arrow), indicating strong invasiveness of the tumor. See **Figure 3**.

### 3.5 Correlation between ultrasound imaging features and GATA3 protein expression

Ultrasound imaging features in BC patients revealed that 65 cases (45.46%) exhibited rich blood flow, 84 cases (58.74%) showed posterior echo attenuation, 100 cases (69.93%) had irregular lesion morphology, 82 cases (57.34%) had unclear lesion boundaries, and 63 cases (44.06%) had a lesion aspect ratio ≥ 1. The longest lesion diameter was (22.29 ± 7.34) mm. GATA3-positive patients showed lower blood flow intensity, posterior echo attenuation, lesion morphology irregularity, and lesion diameter compared to the GATA3-negative group (P < 0.05). Other ultrasound characteristics showed no significant differences between the two groups (P > 0.05). See **Table 3**.

**Table 3:** Correlation between ultrasound imaging features and GATA3 expression ( *x̄* ± s, n%).

Indicator		GATA3			
		Negative (n=75)	Positive (n=68)	T/*x* ^2^ Value	P Value
Blood Flow	Low supply	24 (32.0%)	41 (60.29%)	11.52	0.001
	Rich supply	51 (68.0%)	27 (39.71%)		
Posterior Echo	No attenuation	35 (46.67%)	49 (72.06%)	9.49	0.002
	Attenuation	40 (53.33%)	19 (27.94%)		
Lesion Shape	Regular	32 (42.67%)	57 (83.82%)	25.70	<0.0001
	Irregular	43 (57.33%)	11 (16.18%)		
Lesion Boundary	Clear	37 (49.33%)	44 (64.71%)	3.43	0.06
	Unclear	38 (50.67%)	24 (35.29%)		
Aspect Ratio	<1	34 (45.33%)	39 (57.35%)	2.06	0.15
	≥1	41 (54.67%)	29 (42.65%)		
Lesion Diameter (mm)		22.29 ± 7.34	18.88 ± 6.88	2.86	0.005

### 3.6 Prognostic analysis of 3.0T HR-MRI parameters, ultrasound imaging characteristics, and GATA3 expression in BC patients

Among the 143 breast cancer patients included in this study, 79 (55.2%) were classified as having poor prognosis, defined as the occurrence of metastasis, recurrence, or death during follow-up. Specifically, 78 patients exhibited metastasis, 44 experienced recurrence, and 15 died. The incidence of poor prognosis was significantly higher in GATA3-negative patients compared to GATA3-positive patients (P=0.027). Significant differences in 3.0T HR-MRI parameters were also observed between the groups: patients with poor prognosis had notably higher FA values compared to those with favorable prognosis (P<0.0001), while DA, Dr, and MD values were lower in the poor prognosis group (P<0.0001). Regarding ultrasound imaging characteristics, rich blood supply was more frequently observed in the poor prognosis group (P=0.001), along with more pronounced posterior echo attenuation (P=0.001). Additionally, lesions tended to have irregular shapes (P=0.003) and unclear boundaries (P=0.02) in the poor prognosis group. Although the aspect ratio showed no significant difference between groups (P=0.15), the lesion diameter was slightly larger in the poor prognosis group, approaching statistical significance (P=0.053). These findings indicate that GATA3 expression status, HR-MRI parameters, and ultrasound imaging characteristics are closely associated with the prognosis of BC patients, providing valuable reference points for clinical prognosis assessment. See **Table 4**.

**Table 4:** Comparison of general characteristics between BC patients with poor and favorable prognoses [ *x̄* ± s, n%, M(Q_min_, Q_max_)].

Characteristic		Poor Prognosis Group (n=79)	Good Prognosis Group (n=64)	*x* ^2^/t/z Value	P-Value
GATA3	Negative	48 (60.76%)	27 (42.19%)	4.89	0.027
	Positive	31 (39.24%)	37 (57.81%)		
3.0T HR-MRI Parameters	FA	0.48 ± 0.10	0.41 ± 0.08	4.58	<0.0001
	DA	2.02 (1.92, 2.32)	2.06 (1.94, 2.32)	3.51	<0.0001
	MD	1.21 (1.07, 1.45)	1.28 (1.16, 1.62)	5.30	<0.0001
	Dr	1.08 ± 0.95	1.21 ± 0.10	8.12	<0.0001
Ultrasound Imaging Characteristics					
Blood Flow	Low supply	26 (32.91%)	39 (60.94%)	11.20	0.001
	Rich supply	53 (67.09%)	25 (39.06%)		
Posterior Echo	No attenuation	37 (46.84%)	47 (73.44%)	10.32	0.001
	Attenuation	42 (53.16%)	17 (26.56%)		
Lesion Shape	Regular	36 (45.57%)	45 (70.31%)	8.81	0.003
	Irregular	43 (54.43%)	19 (29.69%)		
Lesion Boundary	Clear	38 (48.1%)	43 (67.19%)	5.25	0.02
	Unclear	41 (51.9%)	21 (32.81%)		
Aspect Ratio	<1	36 (45.57%)	37 (57.81%)	2.12	0.15
	≥1	43 (54.43%)	27 (42.19%)		
Lesion Diameter (mm)		21.73 ± 0.10	19.36 ± 7.19	1.95	0.053

### 3.7 Diagnostic value of combined 3.0T HR-MRI, ultrasound, and GATA3 protein expression for BC prognosis

**Figure 4:**
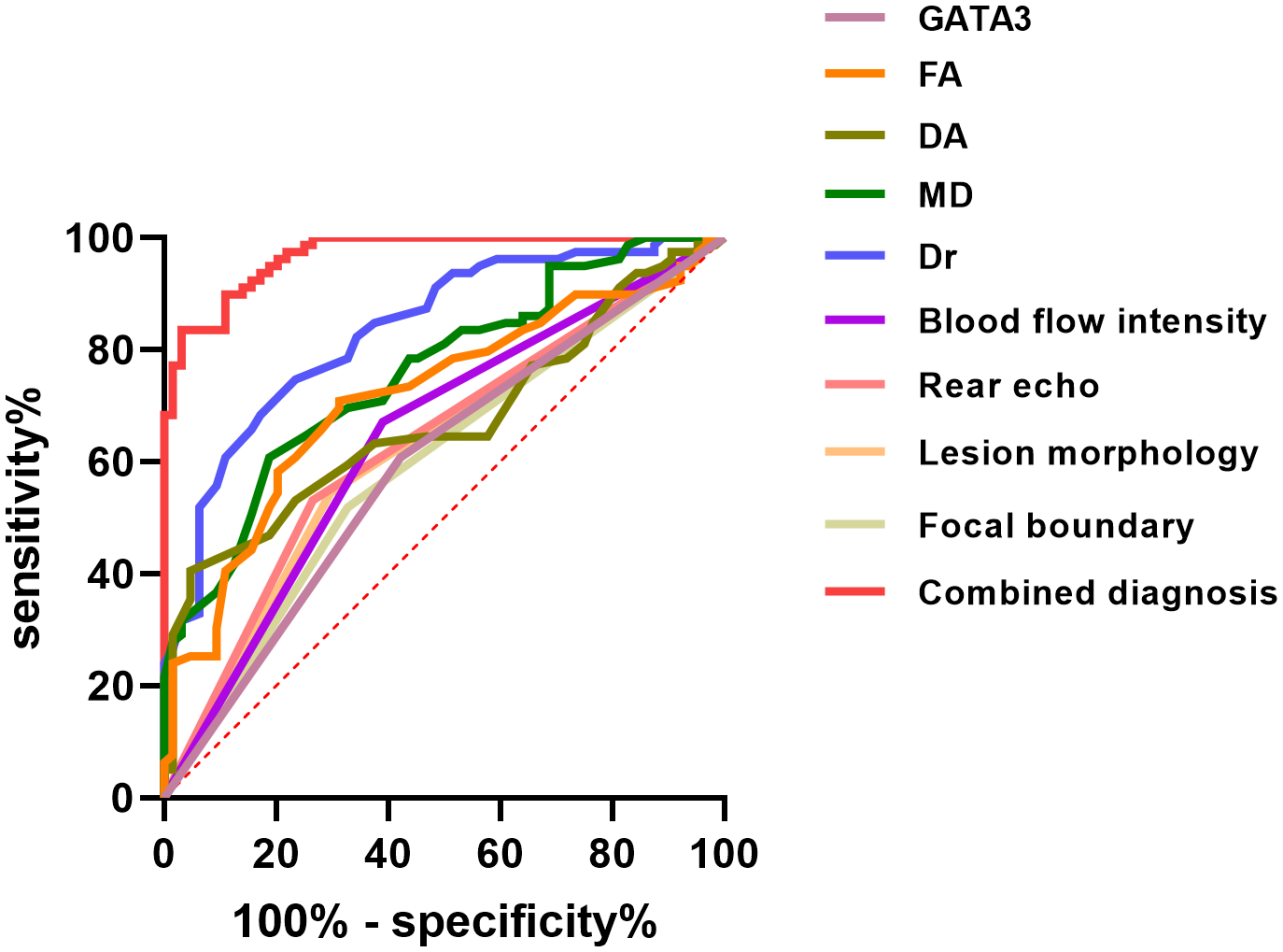
ROC Curve Analysis for Prognostic Evaluation of Breast Cancer.

The results demonstrated that GATA3 expression, 3.0T HR-MRI parameters (FA, DA, MD, and Dr), and ultrasound imaging characteristics (blood flow intensity, posterior echo, lesion shape, and lesion boundary) each have diagnostic value for predicting BC prognosis. The combined diagnostic approach, integrating GATA3, 3.0T HR-MRI parameters, and ultrasound features, yielded the best performance, with an AUC of 0.9695, sensitivity of 83.54%, and specificity of 96.88%. This approach significantly outperformed any single diagnostic parameter, indicating a higher level of diagnostic accuracy and reliability. Therefore, the combined use of GATA3, HR-MRI parameters, and ultrasound imaging features can markedly enhance the diagnostic efficacy for BC, providing more robust diagnostic support for clinical practice. See **Table 5** and **Figure 4**.

**Table 5:** Diagnostic value analysis of combined 3.0T HR-MRI, ultrasound, and GATA3 for BC.

Indicator	AUC	Sensitivity (%)	Specificity (%)	Optimal Cut-off Value
GATA3	0.5929	60.76	57.81	1.440
FA	0.7157	70.89	68.75	0.3964
DA	0.6710	40.51	95.31	0.3582
MD	0.7581	60.76	81.25	0.4201
Dr	0.8347	74.68	76.56	0.5124
Blood Flow Intensity	0.6401	67.09	60.94	1.717
Posterior Echo	0.6330	53.16	73.44	2.001
Lesion Morphology	0.6237	54.43	70.31	1.833
Lesion Boundary	0.5954	51.90	67.19	1.582
Combined Diagnosis	0.9695	83.54	96.88	0.8042

## 4 Discussion

BC is one of the most common malignant tumors in women worldwide, characterized by significant heterogeneity and diverse clinical and pathological features, which increase the complexity of diagnosis and treatment (14). Although existing imaging techniques play an important role in the screening and evaluation of BC, a single diagnostic modality often fails to comprehensively reflect the biological behavior of the tumor (15). Therefore, combining multiple imaging techniques with molecular biomarkers for integrated diagnosis holds significant clinical value (16). For example, Pinker et al. demonstrated that the integration of multiparametric MRI with molecular and genetic markers significantly enhances diagnostic accuracy and enables more personalized treatment planning in breast cancer (17). Similarly, Kepei et al. found that the use of multiparametric breast MRI in conjunction with molecular features improves both diagnostic performance and prognostic assessment compared to single-modality approaches (18). Building on these findings, in this study we conducted a multidimensional evaluation by integrating 3.0T HR-MRI, ultrasound imaging features, and GATA3 protein expression, to explore the clinical utility of this combined approach in BC, particularly in tumor detection and prognostic assessment. Although several studies have examined imaging or molecular features individually, comprehensive investigations that integrate advanced imaging modalities with molecular biomarkers such as GATA3 remain limited. However, recent advances in the field have begun to demonstrate the value of combining imaging-based radiomics with molecular information to improve breast cancer characterization. For example, Wang et al. utilized multidimensional radiomics to enhance the assessment of HER-2 status, providing evidence that such integrative approaches can significantly improve diagnostic accuracy and molecular subtyping in breast cancer (19). These findings highlight the growing recognition that the integration of imaging parameters and molecular markers holds promise for more precise and individualized evaluation. Building on this foundation, our study incorporates 3.0T HR-MRI, ultrasound imaging, and GATA3 protein expression to further extend the scope of multidimensional assessment in breast cancer, particularly in tumor detection and prognostic stratification. To the best of our knowledge, research specifically focusing on the combined diagnostic and prognostic value of 3.0T HR-MRI, ultrasound imaging, and GATA3 expression in breast cancer is still scarce. This highlights the novelty and potential clinical relevance of our approach. Our findings indicate that the combined application of 3.0T HR-MRI, ultrasound parameters, and GATA3 expression shows high potential in improving diagnostic accuracy and prognostic stratification.

GATA3 protein, as a transcription factor associated with mammary epithelial differentiation, plays a crucial role in the development and progression of breast cancer (20). Its expression is closely related to molecular subtypes of the tumor and is also significantly associated with clinicopathological characteristics, and patient prognosis. Assessing GATA3 expression levels can provide a molecular basis for breast cancer subtyping, help predict disease progression and evaluate prognosis, thereby offering a reference for personalized treatment strategies (21). Our study found that the loss or low expression of GATA3 was frequently associated with poor prognosis, whereas positive expression indicated a relatively favorable outcome.

Previous studies have shown that high GATA3 expression is commonly observed in Luminal-type breast cancer and is associated with favorable biological behavior. It has also been suggested that GATA3-positive expression may correspond with more regular ultrasound features, such as well-defined tumor margins and reduced vascular signals (22). However, it is worth noting that systematic analyses exploring the association between GATA3 expression and quantitative MRI parameters remain limited. This observation is consistent with the current literature, where studies investigating the interplay between molecular markers like GATA3 and quantitative imaging parameters are relatively sparse. Our findings, therefore, provide valuable preliminary evidence supporting the added benefit of a multidimensional assessment strategy. As a high-resolution imaging modality, 3.0T HR-MRI can provide detailed insights into tumor tissue structure, cellular diffusion, and microenvironmental status in breast cancer (23, 24). DTI parameters—such as MD, FA, Dr, and Da can reflect changes in cell density, stromal architecture, and diffusion behavior, which in turn indicate alterations in the tumor microenvironment, especially in relation to cellular proliferation and stromal expansion. In our study, GATA3-positive patients demonstrated significantly lower FA values and higher MD, Da, and Dr values, suggesting that their tumor tissues were more structurally intact, with reduced anisotropic diffusion of water molecules—implying lower invasiveness and better prognosis. These imaging findings support the prognostic significance of GATA3 as a favorable biomarker and provide a quantitative imaging correlate for the structural characteristics associated with its expression. While existing research has reported the utility of DTI parameters in tumor grading and Ki-67 prediction (25), systematic analyses combining GATA3 expression and DTI parameters are still in the exploratory stage.

Ultrasound imaging also plays a vital role in the initial screening and classification of breast cancer. B-mode and color Doppler ultrasound can assess tumor size, shape, margin clarity, and posterior echo features, which indirectly reflect tissue composition and vascularity (26, 27). In this study, GATA3-positive patients showed more regular tumor morphology on ultrasound, with preserved posterior echoes, milder echo attenuation, and relatively reduced blood flow signals, suggesting a lower degree of angiogenesis and tissue destruction—consistent with less aggressive biological behavior. Although similar findings have been reported in previous literature, most of them focused on single imaging features. In contrast, our study integrates ultrasound features, HR-MRI parameters, and GATA3 expression to construct a more comprehensive diagnostic framework. Furthermore, the incidence of poor prognosis events was significantly lower in GATA3-positive patients compared to GATA3-negative ones, further supporting the hypothesis of its potential tumor-suppressive role (28). The expression status of GATA3 not only aids in pathological subtyping but may also provide valuable information for predicting recurrence risk and guiding postoperative management (29).

In the prognostic stratification analysis, this study further demonstrated that the combined model incorporating 3.0T HR-MRI parameters, ultrasound imaging features, and GATA3 protein expression exhibited superior accuracy in predicting the prognosis of breast cancer patients. Compared with individual indicators, the combined diagnostic approach achieved an AUC of 0.9695, significantly enhancing diagnostic performance. This finding suggests that the integrating multimodal information can effectively improve our understanding of the biological behavior of breast cancer and provide a more robust data foundation for preoperative assessment and the development of individualized treatment strategies. Compared with previous studies that predominantly focused on a single imaging parameter or molecular marker, our study proposed a more integrated evaluation framework by combining structural imaging (ultrasound), functional imaging (HR-MRI), and molecular biomarkers (GATA3). This approach offers a novel perspective for comprehensive diagnosis and prognostic prediction in breast cancer.

Despite the clinical applicability of this combined assessment strategy, certain limitations remain. First, the study was a single-center retrospective analysis with a relatively small sample size. Future research should further explore the molecular mechanisms of GATA3 in breast cancer to better elucidate the specific association between GATA3 expression and the biological behavior of the disease, thereby providing more robust evidence for personalized treatment.

Overall, this study presents a novel approach to imaging diagnosis and prognosis prediction for breast cancer by integrating multidimensional assessments of 3.0T HR-MRI, ultrasound imaging, and GATA3 protein expression. The combined use of multimodal imaging technologies and molecular biomarkers not only significantly enhances diagnostic sensitivity and specificity but also provides a crucial foundation for developing precise individualized treatment strategies, which may optimize therapeutic outcomes and improve the long-term survival and quality of life for patients. Given the limited number of studies addressing this integrated approach, our research contributes to filling an important gap in the current understanding of breast cancer assessment. Further multi-center and prospective studies are warranted to validate and expand upon these initial findings.

## 5 Conclusion

The combination of 3.0T HR-MRI, ultrasound imaging, and GATA3 protein expression significantly enhances diagnostic accuracy and prognostic evaluation in BC. This comprehensive approach provides valuable insights for developing individualized treatment strategies, with the potential to improve patient outcomes.

## Data Availability

The raw data supporting the conclusions of this article will be made available by the authors, without undue reservation.
